# The European ME/CFS Biomarker Landscape project: an initiative of the European network EUROMENE

**DOI:** 10.1186/s12967-017-1263-z

**Published:** 2017-07-26

**Authors:** Carmen Scheibenbogen, Helma Freitag, Julià Blanco, Enrica Capelli, Eliana Lacerda, Jerome Authier, Mira Meeus, Jesus Castro Marrero, Zaiga Nora-Krukle, Elisa Oltra, Elin Bolle Strand, Evelina Shikova, Slobodan Sekulic, Modra Murovska

**Affiliations:** 1 Institute for Medical Immunology, Charité-Universitätsmedizin Berlin, Campus Virchow, Augustenburger Platz 1/Sudstrasse 2, 13353 Berlin, Germany; 2 August Kirchenstein Institute of Microbiology and Virology, Riga Stradins University, Dzirciema iela 16, Kurzemes rajons, Rīga, 1007 Latvia; 3 Institut de Recerca de la Sida IrsiCaixa-HIVACAT, Institut d’Investigació en Ciències de la Salut Germans Trias i Pujol, IGTP, UAB, Carretera del Canyet, s/n, 08916 Badalona, Spain; 4 Universitat de Vic-UCC, Carrer de la Sagrada Família, 7, 08500 Vic Barcelona, Spain; 5 Deptartment of Earth and Environmental Sciences, University of Pavia, Via Ferrata 7, 27100 Pavia, Italy; 6 Centre for Health Technologies (CHT), University of Pavia, Via Ferrata 5, 27100 Pavia, Italy; 7 Clinical Research Department, Faculty of Infectious & Tropical Diseases, London School of Hygiene & Tropical Medicine, Keppel St, Bloomsbury, London, WC1E 7HT UK; 8 Faculty of Medicine, Paris Est-Creteil University, 8 rue du General Sarrail, 94000 Creteil, France; 9 Pain in Motion International Research Group, Brussels, Belgium; 10 Department of Rehabilitation Sciences and Physiotherapy, Faculty of Medicine and Health Sciences, Ghent University, St. Pietersnieuwstraat 33, 9000 Ghent, Belgium; 11 Department of Rehabilitation Sciences and Physiotherapy (MOVANT), Faculty of Medicine and Health Sciences, University of Antwerp, Prinsstraat 13, 2000 Antwerp, Belgium; 12 Vall d’Hebron University Hospital, CFS/ME Unit, Universitat Autònoma de Barcelona, 119-129, Passeig de la Vall d’Hebron, 08035 Barcelona, Spain; 13 Facultad de Medicina, Universidad Católica de Valencia, San Vicente Mártir, Carrer de Quevedo, 2, 46001 Valencia, Spain; 14 Instituto Valenciano de Patología (IVP) de la Universidad Católica de Valencia San Vicente Mártir, Centro de Investigación Príncipe Felipe (CIPF), Carrer d’Eduardo Primo Yúfera, 3, 46012 Valencia, Spain; 15 Division of Medicine, CFS/ME Center, Oslo University Hospital, Aker, Trondheimsveien 235, 0586 Oslo, Norway; 16 Department of Paediatrics, Norwegian National Advisory Unit on CFS/ME, Rikshospitalet, Sognsvannsveien 20, 0372 Oslo, Norway; 17 Department of Virology, National Center of Infectious and Parasitic Diseases, 44A General Stoletov blvd., 1233 Sofia, Bulgaria; 18 Department of Neurology, Medical Faculty Novi Sad, Hajduk Veljkova 3, 21000 Novi Sad, Serbia

**Keywords:** Biomarker, ME/CFS, European network, Landscape project, Diagnostic, Autoantibodies, Autoimmunity, B cell, Cytokines, Viral

## Abstract

Myalgic encephalomyelitis or chronic fatigue syndrome (ME/CFS) is a common and severe disease with a considerable social and economic impact. So far, the etiology is not known, and neither a diagnostic marker nor licensed treatments are available yet. The EUROMENE network of European researchers and clinicians aims to promote cooperation and advance research on ME/CFS. To improve diagnosis and facilitate the analysis of clinical trials surrogate markers are urgently needed. As a first step for developing such biomarkers for clinical use a database of active biomarker research in Europe was established called the ME/CFS EUROMENE Biomarker Landscape project and the results are presented in this review. Further we suggest strategies to improve biomarker development and encourage researchers to take these into consideration for designing and reporting biomarker studies.

## Biomarker in ME/CFS

Although the exact pathogenesis of myalgic encephalomyelitis or chronic fatigue syndrome (ME/CFS) is still unknown, the most plausible hypothesis is that it is a complex multifactorial syndrome in which immunological and environmental factors play a crucial role. In addition, the severe fatigue, post-exertional malaise, cognitive impairment, and autonomic dysfunction that delineate the disease point to the involvement of both the nervous system as well as metabolic disturbances [1]. Infection by various pathogens, including herpes viruses and enteroviruses, but also intracellular bacteria, are known as triggers of disease. The complex clinical picture and the disagreement on potential pathomechanisms make ME/CFS a controversial entity and compel the research for disease biomarkers that could aid in the diagnostic and clinical management. Biomarker per definition may include both markers with a certain sensitivity and specificity for diagnosing ME/CFS as well as those which may allow to classify subtypes of the disease, be of value as indicators of prognosis, and to be predictive for response to treatment [2].

## The EUROMENE ME/CFS Biomarker Landscape project

EUROMENE is a network of researchers and clinicians from 17 European countries and one COST (Cooperation in Science and Technology) near neighbor country on ME/CFS supported by the European COST program within Horizon 2020 (http://www.cost.eu/COST_Actions/ca/CA15111).

The aims of EUROMENE are to foster strategies for collaboration and harmonization of diagnosis and research, and to compile an inventory of clinical and scientific data in ME/CFS. The Biomarker working group will also try to develop guidelines for the usage of biomarkers and synchronization of biomarker research.

As a first step, a database for active biomarker research in Europe was established called the EUROMENE ME/CFS Biomarker Landscape project. To achieve this, EUROMENE members performed a search for publications on biomarkers within their countries. The search strategy used the medical subject headings (MeSH) term “chronic fatigue syndrome”, which includes myalgic encephalomyelitis, and the respective country, and selected all publications from the last 5 years (2012–2016). The searches were reviewed by members of the biomarker working group. Studies not involving patients with ME/CFS, non-biomarker, and sole treatment studies were excluded, only one review article was included.

**Fig. 1:**
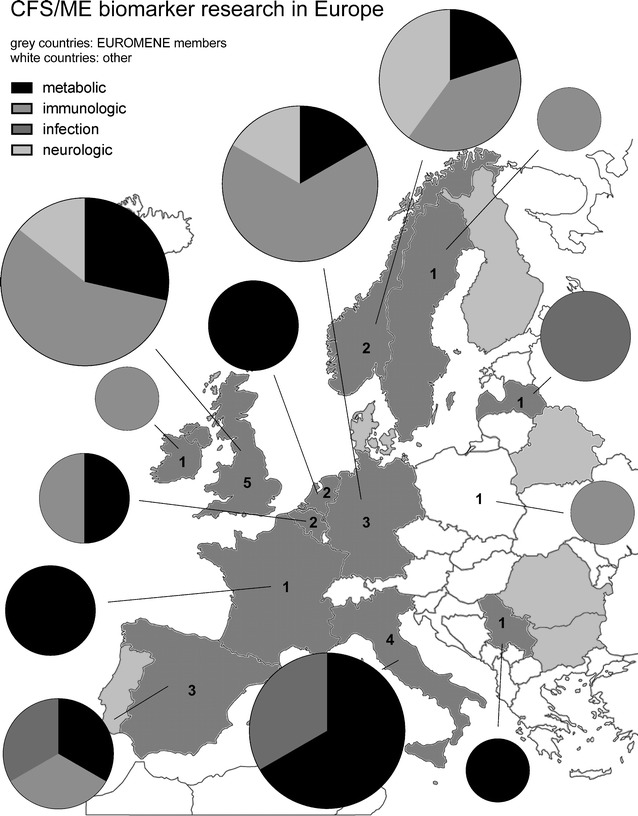
Biomarker studies were categorized as metabolic, immunological, neurological or infection-associated. The data was visualized as total numbers of studies (size of cake) per category (piece of cake) from each country, and the numbers of active biomarker research groups is indicated in the countries. EUROMENE countries are indicated by *grey* (*dark grey* countries with published studies, *light grey* those without studies) and non-EUROMENE by *white*

A total number of 39 studies were identified. Studies were categorized as being immunological, infection-related, metabolic or neurological. We summarize the findings in Fig. 1, which shows the number and type of studies identified in each country, represented by pie charts—their sizes being proportional to the number of identified studies, and their pieces representing the distinct categories of the studies. The number of research groups working on ME/CFS biomarkers in the EU countries is also illustrated in Fig. 1. Countries from which no publications on ME/CFS biomarker could be retrieved are shown in light green/grey, and European countries not participating in the EUROMENE are shown in white. The references listed per countries are shown in Table 1.

**Table 1:** ME/CFS biomarker studies in Europe 2012–2016

Country	Category	Study references
Belgium	Metabolic	[27]
	Immunologic	[3]
France	Metabolic	[28, 29]
Germany	Metabolic	[30]
	Immunologic	[4–7]
	Neurologic	[23]
Ireland	Immunologic	[8]
Italy	Metabolic	[31–34]
	Infection	[18, 19]
Latvia	Infection	[20, 21]
Netherlands	Metabolic	[35, 36]
Norway	Metabolic	[37]
	Immunologic	[9, 10]
	Neurologic	[24, 25]
Poland*	Immunologic	[11]
Serbia	Metabolic	[38]
Spain	Metabolic	[39]
	Immunologic	[12]
	Infection	[22]
Sweden	Immunologic	[13]
UK	Metabolic	[40, 41]
	Immunologic	[14–17]
	Neurologic	[26]

* Non-EUROMENE country

Studies on immune markers (n = 15) in ME/CFS explored immunoglobulins, autoantibodies, cytokines, and immune cell phenotype and function (summarized in Table 2) [3–17]. Four of 5 of the studies on ME/CFS-associated infection markers were focused on XMRV and confirmed the absence of this virus in European ME/CFS cohorts [18–22]. Neurological biomarker studies (n = 4) focused on neurotransmitter regulation, but excluded imaging and functional studies [23–26]. The papers which could be retrieved for potential metabolic markers (n = 15) studied mitochondrial dysfunction, oxidative stress, cortisol regulation, and more comprehensive metabolic pathways [27–41].

**Table 2:** Immune marker studies

Marker^(Ref)^	Design of study	ME/CFS pat. n/diagnostic criteria	Controls n/age- and sex-matched	Sub group analysis	Validation cohort	Results in ME/CFS compared to healthy controls
Immunoglobulins (Ig), MBL [4]	Confirmatory	300/CCC	Reference range	Yes	168	25% diminished Ig 25% elevated Ig 15% MBL diminished
B cells [4]		65/CCC	20/no		20	B cell subsets not altered
IgG3 IgE COMT [5]	Exploratory	76/CCC	74/no	Yes	No	COMT rs4680 is associated with IgG3 and IgE levels
EBV-specific IgG EBV-B and T cells [7]	Confirmatory Exploratory	63/CDC 17	57/no 12/no	Yes	387 No	More EBNA-IgG neg. More VCA-IgM pos EBV B-/T cells lower
HSP60 auto-antibodies [13]	Exploratory	69/CCC	76/no	Yes	61	Few IgG epitopes specific for ME/CFS
Neurotransmitter-receptor auto-antibodies [6]	Exploratory/confirmatory	268/CCC	108/yes	Yes	No	Elevated β2 adrenergic, M3/4 cholinergic receptor antibodies in a subset of ME/CFS
Cytokines [10]	Exploratory	120/CDC	68/yes	Yes	No	Multiple cytokines no differences
Cytokines [8]	Exploratory	48/CDC	35/no	No	No	Elevated CRP, TNF-alpha and IL-6 levels
Cytokines [15]	Review	38 papers				TGF-β levels elevated in 5 of 8 studies (63%)
Cytokines [3]	Exploratory	16/CDC	14/yes	No	No	Increase of IL-1b, IL-8, IL-10 and TNF-alpha levels
BAFF, APRIL [9]	Exploratory	70/CCC & CDC	56/no	Yes	No	Elevated BAFF baseline APRIL not altered
T cells [11]	Exploratory	139/CDC	40/no	Yes	No	Increased CD38 expression on CD8^+^ T cells
NK, T and B cells [12]	Exploratory	22/CDC	30/no	No	No	Treg higher, Tem lower, NK cell CD69, NKp46 higher, CD25 lower, B cell subsets not altered
B cells [16]	Exploratory	38/CCC & CDC	32/yes	No	No	Increased CD24 expression on total B cells Elevated number of CD21+ CD38− B cells
B cells [14]	Exploratory	33/CCC & CDC	24/yes	No	No	Increased number of naïve and transitional B cells
miRNA in immune cell subsets [17]	Exploratory	35/CCC & CDC	50/no	No	No	34 miRNAs upregulated in NK, B cells and monocytes

Diagnostic criteria: *CDC* the Centers of Disease Control or Fukuda Criteria [49], *CCC* Canadian Consensus Criteria

## Discussion

So far there is no single biomarker available for diagnostic use in ME/CFS. Most studies identified here were exploratory in design and lack sex and age-matched control groups or validation cohorts thus having a low evidence level as summarized for the immune marker studies in Table 2 [42]. Some studies report inconsistent data, too. For example an expansion of transitional and naïve B cells and reduced plasmablast levels was reported in one study [14], but could not be confirmed in two other studies [4, 12]. Immune cell phenotype and function analyses are, of course, hampered by variations in sampling and methodological differences between laboratories as most flow cytometric assays are not standardized. Further, immunological biomarkers reported mostly show alterations in subgroups only or with wide overlap to healthy control groups. Such heterogeneous results may be related to the fact that subgroups of ME/CFS patients exist with different immunological pathomechanisms. This concept is supported by the existence of clinical subgroups with heterogeneity in disease onset (infection- versus non-infection triggered), the variability of immune-associated symptoms, and the divergent response to B cell depletion therapy [43]. Research activity in infection markers on ME/CFS across Europe is sparse; however, there is currently no evidence from the available literature that there is a specific serological signature aiding in diagnosis of ME/CFS.

Similar to immunological markers, there is no single neurological or metabolic marker with sufficient specificity and sensitivity as a tool in ME/CFS diagnosis yet. However, recent studies analyzing multiple metabolites could show specific alterations in the majority of ME/CFS patients [37, 44–46] pointing to a probably common and specific metabolic profile. Further, metabolic studies consistently revealed different gender-related patterns [37, 44, 46]. Thus, instead of searching single markers fitting for diagnosing all patients, multiplexed determinations of biomarkers analyzing pathways together with patient stratification, may be necessary to develop diagnostic assays with sufficient sensitivity and specificity [47].

## Conclusions

Heterogeneity of biomarker studies with different case definitions, low number of patients, lack of matched control groups, missing validation studies and potentially subgroup heterogeneity are possible reasons why no diagnostic biomarkers are available yet. Further, as result of the low amount of funding in CFS/ME research few and often small studies were performed so far. Therefore, strategies to improve the quality and to facilitate the comparability of biomarker studies are needed (summarized in Table 3). This starts with well-defined patient cohorts using strict case definitions [47], standardized and quantitative symptom assessment for subgroup analyses, well-defined age- and sex-matched controls, and large enough cohort size and a predefined hypothesis to power the statistical analysis. Detailed description of cohorts, assays performed and results achieved are important to facilitate confirmation studies. Reproducing results in cohorts from different countries, developing Standard Operating Procedures (SOPs) for assays, and multi-center studies are important steps for evaluating the suitability of biomarkers of interest as diagnostic markers. The building of translational networks of clinical and basic research groups like promoted in EUROMENE is an important first step to achieve such goals. Finally, to promote research it is crucial to increase funding for ME/CFS which is currently still far below the budget funds for most other serious diseases in both the EU and the US funding agencies, such as the National Institutes of Health (NIH) [48].

**Table 3:** Strategies for development of diagnostic biomarkers in ME/CFS

1. Standardization of sample collection and assay procedures
2. Use of an uniform clinical case definition
3. Use of questionnaires to assess symptoms and severity to define subgroups
4. Stratification of patients according to sex, disease onset, and disease duration
5. Include sex- and age-matched control groups
6. Sufficient sample size and predefined hypotheses (statistical power)
7. Confirmation of results in validation and multi-center cohort studies
8. Study combinations of biomarkers, perform pathway analysis or functional studies

## Abbreviations

AB: antibody
APRIL: a proliferation-inducing ligand
BAFF: B-lymphocyte activating factor
CCC: Canadian Consensus Criteria
CD: cluster of differentiation
CDC: Fukuda Criteria (Centers for Disease Control and Prevention)
CFS: chronic fatigue syndrome
COMT: catechol-*O*-methyltransferase
COST: Cooperation in Science and Technology
CRP: C-reactive protein
EBNA: EBV nuclear antigen
EBV: Epstein–Barr virus
EU: European Union
EUROMENE: European Network on Myalgic Encephalomyelitis/Chronic Fatigue Syndrome
HSP: heat shock protein
Ig: immune globuline
IL: interleukine
MBL: mannose binding lectin
ME: myalgic encephalomyelitis
MeSH: medical subject headings
NIH: National Institutes of Health
NK: natural killer cells
RNA: ribonucleic acid
SOP: standard operating procedure
TGF: transforming growth factor
TNF: tumor necrosis factor
US: United States of America
VCA: viral capside antigen
XMRV: xenotropic murine leukemia virus-related virus
